# Early mobilization practices of mechanically ventilated patients: a 1-day point-prevalence study in southern Brazil

**DOI:** 10.6061/clinics/2018/e241

**Published:** 2018-10-25

**Authors:** Paula Caitano Fontela, Thiago Costa Lisboa, Luiz Alberto Forgiarini-Júnior, Gilberto Friedman

**Affiliations:** I Programa de Pos-Graduacao em Ciencias Pneumologicas, Faculdade de Medicina, Universidade Federal do Rio Grande do Sul, Porto Alegre, RS, BR; II Serviço de Medicina Intensiva, Hospital de Clinicas de Porto Alegre, Porto Alegre, RS, BR; III Rede Institucional de Pesquisa e Inovacao em Medicina Intensiva, Complexo Hospitalar Santa Casa de Porto Alegre, Porto Alegre, RS, BR; IV Programa de Pos-Graduacao em Biociencias e Reabilitacao e Reabilitacao e Inclusao, Centro Universitario Metodista – IPA, Porto Alegre, RS, BR

**Keywords:** Early Mobilization, Mechanical Ventilation, Intensive Care Unit, Physical Therapy, Prevalence, Survey

## Abstract

**OBJECTIVES:**

To assess early mobilization practices of mechanically ventilated patients in southern Brazilian intensive care units (ICUs) and to identify barriers associated with early mobilization and possible complications.

**METHODS:**

A prospective, observational, multicenter, 1-day point-prevalence study was conducted across 11 ICUs and included all mechanically ventilated adult patients. Hospital and ICU characteristics and patients' demographic data, the highest level of mobilization achieved in the 24 hours prior to the survey and related barriers, and complications that occurred during mobilization were collected in the hospital and the ICU.

**RESULTS:**

A total of 140 patients were included with a mean age of 57±17 years. The median and interquartile range was 7 (3-17) days for the length of ICU stay to the day of the survey and 7 (3-16) days for the duration of mechanical ventilation (MV). The 8-level mobilization scale was classified into two categories: 126 patients (90%) remained in bed (level 1–3) and 14 (10%) were mobilized out of bed (level 4–8). Among patients with an endotracheal tube, tracheostomy, and noninvasive ventilation, 2%, 23%, and 50% were mobilized out of bed, respectively (*p*<0.001 for differences among the three groups). Weakness (20%), cardiovascular instability (19%), and sedation (18%) were the most commonly observed barriers to achieving a higher level of mobilization. No complications were reported.

**CONCLUSIONS:**

In southern Brazilian ICUs, the prevalence of patient mobilization was low, with only 10% of all mechanically ventilated patients and only 2% of patients with an endotracheal tube mobilized out of bed as part of routine care.

## INTRODUCTION

Although the etiology of intensive care unit (ICU)-acquired muscle weakness is multifactorial, early interventions implemented in this setting to minimize the loss of muscle mass and poor physical condition associated with prolonged bed rest seem to improve physical outcomes and reduce the impact of critical illness 1,2. There is a growing body of evidence supporting the safety, feasibility, and benefits of early mobilization in mechanically ventilated ICU patients 3-6. Mobilization and exercise have been shown to reduce the duration of mechanical ventilation (MV) and the length of hospital and ICU stay 7,8, improve physical function at hospital discharge, and reduce long-term rates of hospital readmission and mortality 8-10.

Several studies have evaluated the practice of early mobilization in the ICU 11-16. Nevertheless, limited multicenter research studies have evaluated daily mobilization practices among mechanically ventilated patients 17-20. Most such research, including self-report studies and prevalence studies on mobilization in the ICU, was conducted in Australia and New Zealand 14-18,21, the United States 13,20, and Europe 12,19,21, while there is little data on early mobilization practices in ICUs of underdeveloped or developing countries. In Brazil, the only published study on the provision of early mobilization therapy was retrospectively conducted in a single ICU 22. Thus, the aims of this multicenter 1-day point-prevalence study were (a) to assess early mobilization practices in mechanically ventilated patients admitted to southern Brazilian ICUs and (b) to evaluate the barriers to performing early mobilization and complications during mobilization. A portion of the data presented in the current work has been published in abstract form 23.

## MATERIALS AND METHODS

The present study was conducted with the support of the Sociedade de Terapia Intensiva do Rio Grande do Sul (SOTIRGS), which sent e-mail invitations to the coordinators of critical care units belonging to this society. Interested clinicians also replied via e-mail within a maximum of 30 days after receipt and were asked to confirm their consent in participate. The study was approved by the Research Ethics Committee at the Hospital de Clínicas de Porto Alegre (HCPA)/Universidade Federal do Rio Grande do Sul (UFRGS) (no. 1.335.131).

### Subject characteristics

We developed a survey based on the study of Nydahl et al. 24 and on the opinions of the authors of this study. All mechanically ventilated patients aged >18 years admitted to the participating ICUs during a 24-hour period starting at midnight on the day of the survey were included in the study.

The following variables were collected from each patient: demographic data (sex, main reason for MV, length of ICU stay, and duration of MV up to the day of the survey); airway type (endotracheal tube, tracheostomy, or noninvasive ventilation); highest level of mobilization during the 24-hour study period [categorized using a published 8-level ICU mobilization scale 25]; most important barrier to mobilizing patient to a higher level (as perceived by the participating clinician); and most important complication that occurred during mobilization (as perceived by the participating clinician).

### Hospital and ICU characteristics

The survey consisted of a nonhierarchical list of potential response options for questions, with a text-based “other” option. The hospital type was recorded. The ICU characteristics included the following: 1) ICU type; 2) total number of beds; 3) number of beds occupied by mechanically ventilated patients; 4) staffing ratio of practical nurses, nurses, physicians, physical therapists, and occupational therapists; 5) clinician ordering mobilization; 6) staff involved in mobilization; 7) selected protocols; 8) available equipment for mobilization; and 9) other equipment/resources for early mobilization available.

### Survey distribution

The study was conducted from June 20 to 24 (Monday to Friday). Participating clinicians were sent reminders a month and a week prior the upcoming survey. On June 19, one researcher randomly chose which week day the data would be collected by selecting one of the five sealed opaque envelopes. Weekend days were excluded because the mobilization routine on weekends is different due to staff shortages.

Participants received e-mail and cellular text message notifications by 7:00 am on the day after the selected study day with a request to collect data from medical records on the highest activity of mobilization undertaken by patients in the previous 24 hours. This reduced the possibility that previous knowledge about the day of the survey could influence the quality and quantity of early mobilization activities. Participants were asked to complete data collection within 3 days, with access to a 24-hour/day investigator's telephone line to immediately answer any questions. All data variables to be collected were detailed to aid standardization and comparability of data collection among all participants.

To facilitate data collection, participants completed a web-based electronic form created using SurveyMonkey^®^ software, which enabled real-time integration with the Statistical Package for the Social Sciences (SPSS) software.

### Statistical analysis

Normally distributed variables were described as the mean and standard deviation, and asymmetrically distributed variables were described as the median and interquartile range. Proportions were expressed as percentages. The chi-square test and the Fisher exact test were used to evaluate statistical associations. Similar to Nydahl et al. 19, the 8-level mobilization scale was evaluated as a binary variable (“remained in bed” - level 1-3 or “mobilized out of bed” - level 4-8). Data were analyzed using SPSS software, version 18.0. Statistical significance was defined as a *p*-value less than 0.05.

## RESULTS

Data were collected on June 21, 2016, in 11 ICUs by 10 unique physicians (one physician collected data for two ICUs within the same hospital), yielding a sample of 140 patients. The median (interquartile range) number of beds available in the participating ICUs was 16 10-29, 12 6-18 of which were occupied by mechanically ventilated patients and were included in this study.

### Subject and ICU characteristics

The most common hospital and ICU types were university-affiliated hospitals and medical-surgical ICUs (Table 1). Physicians were the most common clinician ordering early mobilization in 10 ICUs. The clinicians who were the most involved in patient mobilization were physical therapists, practical nurses, and nurses. Clinical protocols commonly used in the participating ICUs included standardized sedation and MV weaning. Equipment commonly available within the ICUs to facilitate or promote patient mobilization included portable ventilators, lifting devices, and special beds (Table 2).

Patients included in the study were identified by a code established by the clinician responsible for data collection, and no information that could identify patients was collected. Of the 140 patients included, 64% (n=90) were male, and the mean age was 57±17 years. The median and interquartile range 7 3-17 days for length of ICU stay to the day of the survey and 7 3-16 days for the duration of MV. The main causes for MV were pneumonia/respiratory infection and neurological dysfunctions (Table 4).

### Mobilization

Out-of-bed mobilization was applied in only 14 patients (10%), 60% (n=83) of patients were at most turned in bed, and only 3 patients (2%) stood, marched, or walked on the day of the survey. The distribution of airway types used for ventilation included 70% (n=98) endotracheal tubes, 24% (n=34) tracheostomies, and 6% (n=8) noninvasive ventilation. There was a significant difference in the proportion of patients mobilized out of bed in terms of airway type: 2% endotracheal tubes, 23% tracheostomies, and 50% noninvasive ventilation (*p*<0.001) (Table 3). None of the 98 patients with an endotracheal tube was reported to stand, march, or walk on the day of the survey.

Out-of-bed mobilization was similar among different ICU types (comparing medical, surgical, trauma, and coronary ICUs; *p*=0.065), length of ICU stay (comparing <7 days and ≥7 days; *p*=0.236), duration of MV (comparing <7 days and ≥7 days; *p*=0.176), and age (comparing <60 years and ≥60 years; *p*=0.583).

With regard to causes for MV, acute pulmonary edema and neoplasms were more common in patients mobilized out of bed compared with those who remained in bed (Table 4).

### Barriers and complications to mobilization

Weakness (20%; n=28), cardiovascular instability (19%; n=26), and sedation (18%; n=25) were the most commonly reported barriers to accomplishing a higher level of mobilization. Weakness was the most limiting barrier to mobilization out of bed (Table 5) and in patients with noninvasive ventilation versus tracheostomy versus endotracheal tube (62% *vs*. 50% *vs*. 6%; *p*<0.001). The most commonly reported barriers were sedation and cardiovascular instability for mechanically ventilated patients with an endotracheal tube versus tracheostomy versus noninvasive ventilation (23% *vs*. 6% *vs*. 0%; *p*=0.025 and 25% *vs*. 3% *vs*. 0%; *p*=0.003, respectively). Consciousness impairment was a more common barrier in mechanically ventilated patients with tracheostomy than in patients with an endotracheal tube or noninvasive ventilation (12% *vs*. 1% *vs*. 0%; *p*=0.039). No complications were reported by participants during patient mobilization.

## DISCUSSION

This report represents the first multicenter Brazilian survey on early mobilization during MV. Our study confirms that mobilization out of bed is uncommon, and 60% of patients were only turned in bed. None of the patients with an endotracheal tube stood, marched, or walked on the day of the survey. The most common barriers to mobilization were weakness, cardiovascular instability, and sedation.

No complications were reported for patients who had any level of mobilization, a result in line with the literature showing that mobilization is safe 1,2,7-10,26. Nonetheless, the utilization of early mobilization is low in mechanically ventilated patients, as observed in our study and in previous studies 17,19,20. The prevalence of out-of-bed mobilization in this study was lower than those observed in Germany 19 and the United States 20. However, it was similar to that observed in a study conducted in Australia and New Zealand by Berney et al. 17, which showed that no patient requiring MV was mobilized out of bed. In a prospective multicenter study carried out in the same countries, mobilization occurred in only 16% of 1288 physical therapy sessions with mechanically ventilated patients 18. A retrospective unicenter study by Pires-Neto et al. 22 in Brazil showed that out-of-bed activities occurred in 29% of 1426 mobility therapy sessions, with the highest prevalence in patients with tracheostomy (27%). The reporting of patient characteristics varied between studies, but several variables were similar: age, gender, and reason for ICU admission, among others.

The first study to perform an international comparison on mobilization revealed that Australian patients were more likely to be mobilized and to receive early mobilization than Scottish patients, whereas the latter were more likely to be mobilized while on MV 21.

The type of artificial airway seems to be considered an important barrier. This is supported by data from our study and from other studies that found a significant difference in the proportion of patients mobilized out of bed when comparing noninvasively ventilated, tracheostomized and intubated patients 17,19,20. In a study by Nydahl et al. 19, 8%, 39%, and 53% of patients with an endotracheal tube, tracheostomy, and noninvasive ventilation were mobilized out of bed, respectively. In a two-day point-prevalence study by Jolley et al. 20, MV via an endotracheal or tracheostomy tube were negative predictors of out-of-bed mobilization. Patients with tracheostomy or noninvasive ventilation seem to be easier to mobilize because of the small amount of equipment needed and the lower risk of airway complications during mobilization. However, a systematic review including 13 clinical trials reported only one self-extubation during out-of-bed mobilization, with no need for reintubation 27.

Lower rates of mobilization in intubated patients may be explained by the fact that two of the most perceived barriers are cardiovascular instability and sedation. Intubated and mechanically ventilated patients are usually in a more critical phase of their disease, require deeper sedation, and are more frequently hemodynamically instable than patients with tracheostomy 28 or noninvasive ventilation. However, there was no difference in the proportion of patients mobilized out of bed in terms of length of ICU stay and duration of MV when comparing <7 days and ≥7 days. This reinforces the idea that ventilation with an endotracheal tube in our ICUs is a barrier even in chronic or recovering patients.

The organizational and structural characteristics may also explain the low levels of mobility observed. Previous studies have identified the nurse/patient ratio, physiotherapy staffing and the use of protocols to standardize care as significant predictors of ICU mobility 29,30. Despite the use of protocols, the proportion of ICUs in our study with early mobilization protocols was greater than those reported in France (24%), Germany (30%), the United Kingdom (20%) and the United States (30%) 30. However, the nurse/patient ratio and the physiotherapy team were smaller. In our study, nursing providers were the professionals who were primarily involved in patient mobilization in conjunction with the physiotherapists.

The main barrier to providing patients with a higher level of mobilization was muscle weakness. A recent systematic review of quantitative and qualitative studies identified weakness as an important capability-related barrier to mobilization 31. The low prevalence of mobilization practice and of patients mobilized out of bed observed in our study, regardless of length of ICU stay and duration of MV, may be explained by the difficulty in mobilizing patients when they are in the critical phase of their disease and, subsequently, because they experience the effects of such immobility, creating a cycle of immobility 32. Therefore, weakness should be a key reason why early mobilization is of great importance in the ICU.

Complications, even transient events such as patient-ventilator asynchrony desaturation, or blood pressure changes, were not reported in patients in the present study during mobilization. The incidence of events such as hemodynamic instability or desaturation is very low during mobilization practice, as shown in a meta-analysis of safety by Nydahl et al. 26. However, our findings may be the result of missing data in the patient's medical records. Cases of almost incidents are rarely documented in medical records 33.

Our study has potential limitations. First, the participation of ICUs was voluntary, which means that it is possible that only ICUs with enough staffing or interest in early mobilization participated. This study included only 11 of the 33 ICUs invited to participate; therefore, the sample size was small. Eight of the eleven ICUs were located in the state capital, and 10 were located within university and university-affiliated hospitals. Additionally, the state where the data were obtained has one of the highest human development indexes in Brazil. Moreover, data collection was based on the report of participating physicians and not on direct observation. However, self-reporting is the most realistic method for studies such as this and has been employed in previous studies 7,9,17,19,20.

Second, data collection was based on medical records, and therefore, missing data is possible. However, documentation of out-of-bed patient mobilization seems to be of high priority for documentation 34 and has considerable agreement with observed mobilization in the ICU 35.

Third, to ensure that the survey was included all mechanically ventilated patients with no missing data, some data of interest were not collected, such as sedation level or severity of illness.

Lastly, the interpretation of the ordinal scale among different physicians may have led to disparity in the reporting of mobilization. However, in all phases of this study, participants received assistance on how to interpret and use the scale.

In this 1-day point-prevalence study of 11 ICUs in southern Brazil, we found that 90% of patients were mobilized only in bed, with higher-level mobilization seldomly occurring. Patients with endotracheal tubes were less likely to be mobilized out of bed. The participants identified weakness, cardiovascular instability, and sedation as the main barriers to mobilization out of bed, although some barriers may be modifiable and important reasons to increase mobilization. No complications were reported during patient mobilization.

## AUTHOR CONTRIBUTIONS

Fontela PC designed the study, analyzed the data, wrote the manuscript, reviewed the data analysis and approved the final version of the manuscript. Lisboa TC helped conduct the study, analyzed the data, reviewed the manuscript and approved the final version of the manuscript. Forgiarini-Júnior LA analyzed the data, reviewed the manuscript and approved the final version of the manuscript. Friedman G designed the study, analyzed the data, wrote the manuscript, reviewed the data analysis and approved the final version of the manuscript.

## Figures and Tables

**Table 1:** Characteristics of the participating ICUs (n=11).

Characteristics	n (%)	Total patients enrolled, n (%)	Number of patients included in the study, median (IQR)
Type of hospital			
University	1 (9)	23 (16)	-^a^
University-affiliated^b^	9 (82)	113 (81)	12 (6 – 18)
Community	1 (9)	4 (3)	- ^a^
Type of ICU			
Medical-surgical	8 (73)	103 (74)	12 (6 – 21)
Medical	1 (9)	12 (9)	- ^a^
Trauma	1 (9)	18 (13)	- ^a^
Transplantation	1 (9)	7 (5)	- ^a^
Number of ICU beds, mean ± SD			
ICU beds	18.7±8.9		
ICU beds occupied by mechanically ventilated patients	12.7±6.7		
Staffing ratio, mean ± SD			
Practical nurse to patient	1.8±0.3		
Nurse to patient	7.3±2.6		
Physical therapist to patient	10.0±3.3		
Physician to patient	6.1±2.2		

IQR: Interquartile range;

SD: Standard deviation;

ICU: Intensive care unit;

^a^: Median and IQR not calculated due to the small sample size.

^b^: University-affiliated hospitals have an association with universities but are not operated by a university.

**Table 2:** Mobilization practices, clinical protocols, and equipment available in the participating ICUs.

Characteristics	ICUs (n=11) n (%)
Type of clinician ordering patient mobilization	
Physician	10 (91)
Physical therapist	1 (9)
Staff involved in patient mobilization^a^	
Physician	5 (45)
Physical therapist	11 (100)
Nurse	10 (91)
Practical nurse	11 (100)
Clinical protocols^a^	
Early mobilization	5 (45)
Standardized sedation	7 (64)
Daily interruption of sedation	5 (45)
Evaluation for pain and delirium	4 (36)
Weaning from MV	7 (64)
Synchronized daily wake-up and SBT	4 (36)
Equipment available within the ICU^a^	
Special bed	7 (64)
Special chair	6 (54)
Lifting device	7 (64)
Walker	5 (45)
Sliding board	6 (54)
Portable ventilator	8 (73)
Cycle ergometer	5 (45)
Neuromuscular electrical stimulation	6 (54)
Tilt table	2 (18)
Continuous passive mobilization	1 (9)

ICU: Intensive care unit;

MV: Mechanical ventilation;

SBT: Spontaneous breathing trial;

^a^: More than one response could be provided for each survey question; hence, proportions add to more than 100%.

**Table 3:** Highest level of mobilization achieved on the study day.

Level of mobilization	Total (n=140) n (%)	Airway type		
		Endotracheal tube (n=98) n (%)	Tracheostomy (n=34) n (%)	NIV (n=8) n (%)
Remaining in bed^a^	126 (90)	96 (98)	26 (76)	4 (50)
No mobilization	25 (18)	23 (23)	2 (6)	0 (0)
Turning in bed	58 (41)	45 (46)	13 (38)	0 (0)
Sitting in bed	43 (31)	28 (29)	11 (32)	4 (50)
Mobilized out of bed^a^	14 (10)	2 (2)	8 (23)	4 (50)
Sitting on edge of bed	2 (1)	1 (1)	1 (3)	0 (0)
Sitting out of bed	9 (6)	1 (1)	5 (15)	3 (37)
Standing out of bed	1 (1)	0 (0)	0 (0)	1 (12)
Marching in place	1 (1)	0 (0)	1 (3)	0 (0)
Walking	1 (1)	0 (0)	1 (3)	0 (0)

NIV: Noninvasive ventilation;

^a^: *p*<0.001 according to the chi-square test comparing airway type for remaining in bed *versus* mobilized out of bed.

**Table 4:** Main cause of MV.

Cause for MV	Total (n=140) n (%)	Remaining in bed (n=126) n (%)	Mobilized out of bed (n=14) n (%)	*p*^a^
Pneumonia/respiratory infection	35 (25)	34 (27)	1 (7)	0.189
Neurological disorder	31 (22)	29 (23)	2 (14)	0.735
Postoperative complications	15 (11)	15 (12)	0 (0)	0.363
ARDS	13 (9)	11 (9)	2 (14)	0.620
Trauma	11 (8)	9 (7)	2 (14)	0.602
COPD	9 (6)	7 (6)	2 (14)	0.222
Cardiac arrest	8 (6)	8 (6)	0 (0)	>0.999
Heart failure	3 (2)	3 (2)	0 (0)	>0.999
Acute pulmonary edema	3 (2)	1 (1)	2 (14)	0.026
Septic shock	3 (2)	3 (2)	0 (0)	>0.999
Neoplasm	3 (2)	1 (1)	2 (14)	0.026
Other	6 (4)	5 (4)	1 (7)	0.475

MV: Mechanical ventilation;

ARDS: Acute respiratory distress syndrome;

COPD: Chronic obstructive pulmonary disease;

^a^: Calculated using the Fisher exact test.

**Table 5:** Perceived barriers to achieving a higher level of mobilization.

Barriers	Total (n=140) n (%)	Remaining in bed (n=126) n (%)	Mobilized out of bed (n=14) n (%)	*p*^a^
Weakness	28 (20)	17 (13)	11 (79)	<0.001
Cardiovascular instability	26 (19)	25 (20)	1 (7)	0.467
Sedation	25 (18)	25 (20)	0 (0)	0.075
Restlessness	14 (10)	12 (9)	2 (14)	0.633
Palliative care	11 (8)	11 (9)	0 (0)	0.602
Endotracheal tube	5 (4)	5 (4)	0 (0)	>0.999
Consciousness impairment	5 (4)	5 (4)	0 (0)	>0.999
Intracranial hypertension	5 (4)	5 (4)	0 (0)	>0.999
Paraplegia	4 (3)	4 (3)	0 (0)	>0.999
Renal replacement therapy	3 (2)	3 (2)	0 (0)	>0.999
Heavy MV	3 (2)	3 (2)	0 (0)	>0.999
Respiratory rate	2 (1)	2 (2)	0 (0)	>0.999
Unstable SCI	2 (1)	2 (2)	0 (0)	>0.999
Bronchospasm	2 (1)	2 (2)	0 (0)	>0.999
Pain	1 (1)	1 (1)	0 (0)	>0.999
Delirium	1 (1)	1 (1)	0 (0)	>0.999
Presence of drains	1 (1)	1 (1)	0 (0)	>0.999
Cardiac arrest on the day of the survey	1 (1)	1 (1)	0 (0)	>0.999
Obesity	1 (1)	1 (1)	0 (0)	>0.999

MV: Mechanical ventilation;

SCI: Spinal cord injury;

^a^: Calculated using the Fisher exact test.
